# Assessing Alloimmunization in Sickle Cell Disease Patients: Insights From a Tertiary Care Center Study on Multiple Blood Transfusion Recipients in Central India

**DOI:** 10.7759/cureus.92371

**Published:** 2025-09-15

**Authors:** Girish Singh Kshatriya, Sankalp Sharma

**Affiliations:** 1 Transfusion Medicine, All India Institute of Medical Sciences, Raipur, Raipur, IND; 2 Blood Bank, Government Medical College Jagdalpur, Jagdalpur, IND

**Keywords:** alloimmunization, haemolytic anemia, rh blood group, sickle cell disease, transfusion medicine

## Abstract

Introduction: Alloimmunization is defined as a delayed transfusion reaction, which occurs days to months after transfusion, in which a patient may produce antibodies against a red cell antigen that the patient lacks. This alloimmunization process is influenced by genetic as well as acquired patient-related factors. This study aims to determine the pattern and frequency of allo-antibodies in patients with sickle cell disease (SCD) who have undergone multiple transfusions.

Material and methods: We carried out a prospective observational study at a tertiary care hospital in central India. Our focus was on patients with SCD who received multiple blood transfusions to assess their alloimmunization status by using Panoscreen® I, II, and III (Immucor, Inc., Norcross, GA) and PANOCELL 16 (Immucor, Inc.) antibody identification panel.

Results: Among 140 samples screened, five (3.57%) tested positive for antibodies. Of these, two (1.42%) had autoantibodies and three (2.15%) had alloantibodies. The prevalence of alloimmunization was not statistically significant (p = 0.418, 95% CI).

Conclusion: Alloantibodies detected were predominantly against the Rh system and not clinically significant. However, the findings highlight the need for enhanced immunohematologic testing to prevent alloimmunization and improve transfusion care in SCD patients.

## Introduction

Sickle cell disease (SCD) is a monogenetic disorder caused by a point mutation in the beta globin gene, leading to the replacement of glutamic acid with valine at the sixth position of the beta globin chain of the hemoglobin molecule [1]. Hemoglobinopathies are the most prevalent genetically inherited disorders affecting red blood cells worldwide [2]. Globally, over 42 million carriers and more than 12,000 affected infants are added annually to the hemoglobinopathy burden [2]. In India, the cumulative gene frequency of hemoglobinopathies is around 4.2% [3]. SCD is widespread in central India, particularly in Chhattisgarh, where it significantly impacts health and well-being. In fact, around 10% of the population, approximately 2.5 million people, are estimated to carry the SCD allele, highlighting the urgent need for awareness and support for those affected by this genetic disorder [4]. Of these, approximately 10% or 250,000 individuals are homozygous for the allele and suffer from SCD [5]. Patra et al., in their study of four years in three lakh people, have reported 9.3% of sickle cell trait and 0.21% of SCD in a screening program for the sickle cell gene in Chhattisgarh [5]. Despite the availability of hydroxyurea, the need for chronic transfusions has risen in recent years. Transfusions help reduce morbidity and mortality, and in children, they prevent the first stroke [6]. However, repeated blood transfusions can result in erythrocyte alloimmunization, which poses significant complications for the patient [7]. These antibodies are often directed against antigens expressed on RBCs that may cause a transfusion reaction in subsequently transfused units expressing the corresponding red cell antigen [7]. Finding compatible blood units for alloimmunized patients can be challenging and time-consuming, often leading to transfusion delays, especially in acute situations. Alloimmunization can occur from pregnancy, transfusions, transplantation, needle sharing, or exposure to immunogenic materials. Healthy blood donors exhibit very low rates of immunization, whereas in chronically transfused patient populations, such as those with sickle cell anemia or thalassemia, alloimmunization rates may range from 14% to 50% [8]. A clinically significant red cell antibody is linked to hemolytic disease of the fetus and newborn (HDFN), transfusion reactions, or reduced red cell survival. Their clinical significance varies, with some causing rapid destruction of incompatible red cells and others having a minimal or negligible impact on survival. The SCD patient population has a high alloimmunization prevalence, ranging from 7% to 59%, compared to just 2% to 3% in sporadically transfused patients from general hospital populations [9-12]. While partially phenotypically matched blood products reduce alloimmunization, they do not completely prevent it. Regular screening of multi-transfused patients should be conducted to detect any alloimmunization [13]. The most effective prevention method is to transfuse genotype-matched red blood cells. While genotype-matched transfusions remain the most effective preventive strategy, cost and feasibility constraints in low-resource settings like India necessitate optimizing phenotype-matching strategies tailored to local demographics [14].

The primary antigens associated with alloimmunization in the Indian population include D, Rh subgroups, E, e, C, c, K, k, Jka, Jkb, Fya, Fyb, S, s, M, N, Lea, and Leb [15,16]. Most studies on alloimmunization in SCD patients have reported that alloantibodies are commonly formed against Rh, Kell, and Duffy antigens [16].

Utilizing partially phenotypically matched red cells for Rh (D, C, c, E, e) and Kell antigens significantly reduces the incidence of alloimmunization in SCD, with rates dropping from 30% to below 3% [15]. Extending antigen matching to include the Kidd, Duffy, and MNS systems has been shown to further reduce the risk of alloimmunization [17]. Alloantibodies can reduce the survival of transfused RBCs and cause delayed hemolytic transfusion reactions (DHTRs) days to weeks later, affecting an estimated 1.6% to 11% of SCD transfusion patients, who may experience increased fatigue, jaundice, dark urine, fever, and pain [18,19]. The term hyperhemolysis refers to DHTRs in patients with SCD, where severe hemolysis results in hemoglobin levels dropping below pre-transfusion values [20]. This indicates that the patient's own red blood cells are being hemolyzed in addition to the transfused cells [21]. Severe hemolysis can occur in the absence of identifiable antibodies and with a negative direct antiglobulin test (DAT) [22].

Aim & objectives

Aim

To screen multi-transfused SCD patients for the detection of alloantibodies and provide the allo-antibody status of SCD patients registered at a tertiary care hospital during the study period.

Objectives

Study the prevalence of alloantibodies among SCD patients with multiple blood transfusions. To determine the pattern and frequency of alloantibodies in multi-transfused SCD patients. To identify and characterize any autoantibodies or indeterminate reactions (such as pan-agglutination) observed during antibody screening.

## Materials and methods

The study of multiple blood transfusion recipient SCD patients for alloimmunization status was conducted at the Department of Transfusion Medicine of a tertiary care hospital in Chhattisgarh, central India, between March 2019 and July 2020. This study was carried out as per the approved protocol after obtaining clearance from the institutional scientific committee and ethics committee.

Inclusion criteria

Multi-transfused SCD patients, aged between one and 65 years, diagnosed with SCD, who had received two or more units of whole blood or red cell concentrate at a tertiary care hospital in Chhattisgarh, were included in the study.

Exclusion criteria

Exclusion criteria included patients with SCD who had received fewer than two transfusions of whole blood or packed red blood cells in their lifetime; routine transfusions not tested with high-performance liquid chromatography (HPLC) or associated with undiagnosed SCD; and patients diagnosed with hemoglobinopathies other than SCD.

Sample size calculation

The sample size was calculated with the help of the Department of Biostatistics using the Cochran formula:

n = {(Z²) × P × Q} / e².

Where Z = 1.96 (95% confidence level), P = prevalence (10% prevalence of SCD in Chhattisgarh) [4], Q = (100 - P) = 90%, and e = margin of error (5%). By substituting the values, the calculated sample size was 138.29, rounded to 140 cases.

Documentation and sample collection

Clinical history and examination details were obtained. Previous HPLC reports were documented. Written informed consent was obtained from all multi-transfused SCD patients included in the study; for patients <18 years, consent was obtained from a parent or legal guardian. Patient details were recorded using a standardized proforma.

Blood samples received in the Department of Transfusion Medicine and Blood Bank for crossmatching were collected in 5 mL K₂ or K₃ ethylenediaminetetraacetic acid (EDTA) tubes (lavender top) for plasma separation, or plain tubes (red top) for serum separation, to ensure adequate volume and correct labeling. Samples were centrifuged, plasma/serum separated, and serum/plasma samples were aliquoted and stored at -40°C until antibody screening was performed.

Laboratory evaluation

**Figure 1:**
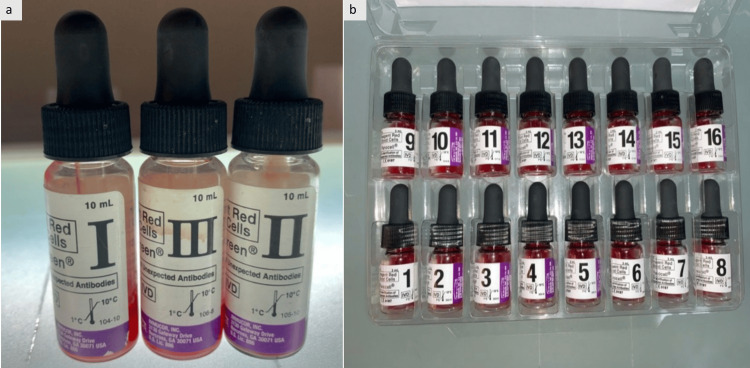
(a) Panoscreen® I, II, and III (reagent cells) (Immucor, Inc.) and (b) PANOCELL 16 (Immucor, Inc.) antibody identification panel (reagent cells).

In this tertiary care hospital, 100% partial antigen matching for transfusion in SCD patients is not routinely practiced; transfusions are primarily based on ABO and RhD matching. All blood samples were subjected to antibody screening by the indirect antiglobulin test (IAT) using the gel card method. Screening was performed with a commercial three-cell panel (Bio-Rad, Hercules, CA/Immucor, Inc., Norcross, GA) (Figure 1A). Samples testing positive were further analyzed using a 16-cell antibody identification panel (Bio-Rad/Immucor, Inc.) (Figure 1B).

Data were compiled in Microsoft Excel 2019 (Microsoft Corporation, Redmond, WA) and analyzed using SPSS version 25 (IBM Corp., Armonk, NY). Descriptive statistics, such as frequencies and percentages, were calculated. To assess the association, Fisher's exact test was applied. A p-value of < 0.05 was considered statistically significant, and 95% confidence intervals were calculated.

## Results

**Figure 2:**
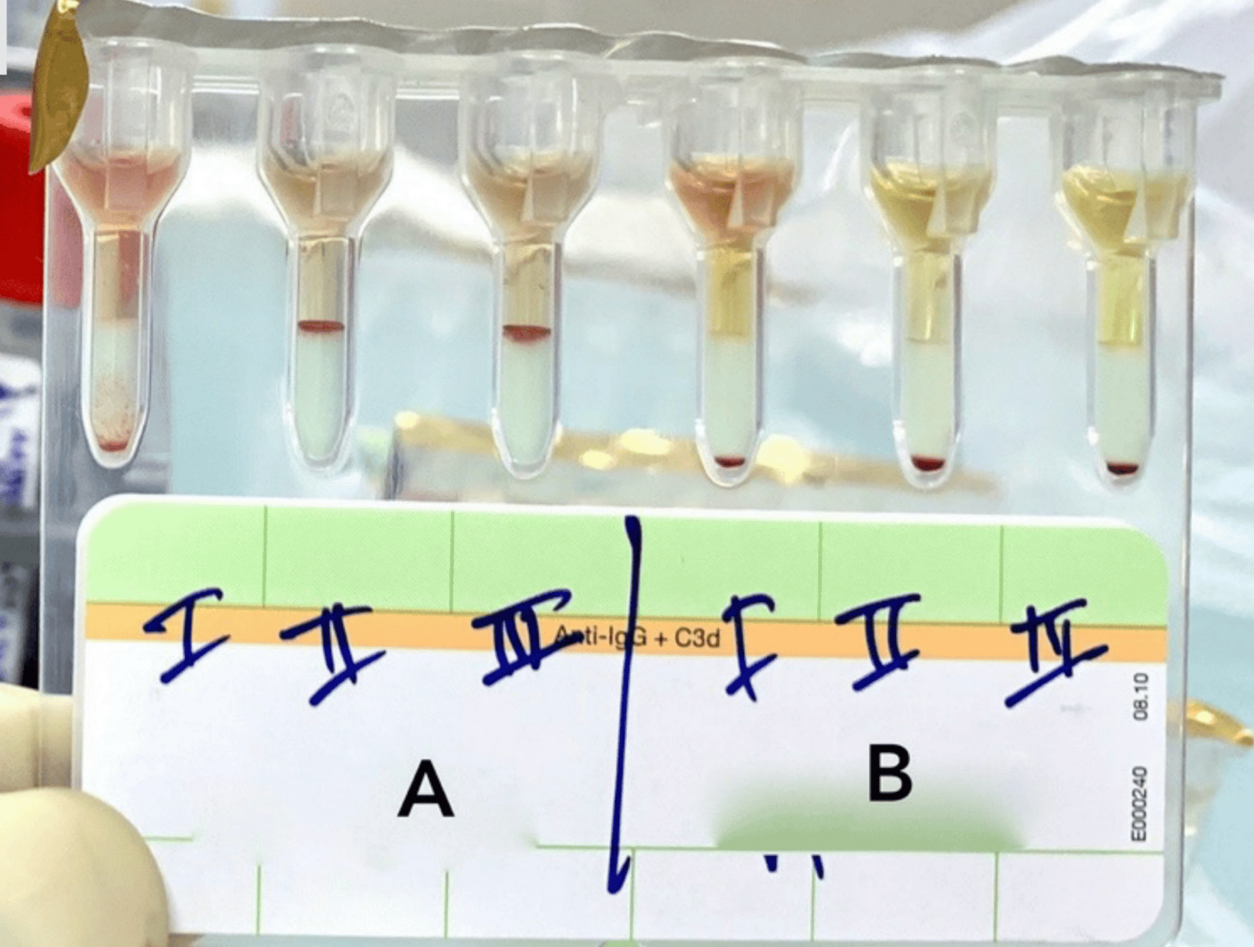
Results of two samples, denoted by A & B, tested for antibody screening using a poly-specific Coombs card and three-cell panel (Panoscreen). Result for sample A showing 1+ reaction in microtube I and 4+ reaction in microtubes II and III. For sample B, microtubes I, II, and III showed negative reaction.

Out of 140 patients, including 75 males and 65 females (male-to-female ratio = 1.15:1), the percent-wise distribution was 46.4% females and 53.6% males. Out of 140 subjects, 42 were of Sahu ethnicity; the ethnicities of other patients could not be traced. A total of 133 subjects were residents of Chhattisgarh, and seven were from outside of Chhattisgarh. Out of 140 patient samples studied, 34 had “A” blood group, which is 24.3% of total cases, 11 had “AB” blood group, which is 7.9% of total cases, 51 had “B” blood group, which is 36.4% of total cases, and 44 had “O” blood group, which is 31.4% of total cases. Two patients out of 140 were Rh “negative”, and the rest 138 were Rh “positive”. The mean hemoglobin (Hb) level of the study sample was 6.0 g/dl, with a minimum Hb of 1.7 g/dl and a maximum Hb of 14.3 g/dl. A positive RBC antibody screening result was obtained in five patients (3.57%) (Table 1 and Figure 2).

**Table 1:** Antegram of three-cell panel Panoscreen (Immucor, Inc.)/antigen phenotyping of the screening cells.

		Rh-Hr							Kell						Duffy		Kidd		Lewis		P	MN				Lutheran		Xg	Patient’s test results						
CELL	Donor	D	C	c	E	e	Cw	K		k	Kpa	Kpb	Jsa	Jsb	Fya	Fyb	Jka	Jkb	Lea	Leb	P1	M	N	S	s	Lua	Lub	Xga	CELL						
1	R1R1 B6595	+	+	0	0	+	0	0		+	0	+	0	+	0	+	+	0	+	0	0	0	+	0	+	0	+	0	1						
2	R2R2 C2158	+	0	+	+	0	0	0		+	0	+	0	+	+	0	+	+	0	+	+	+	0	0	+	0	+	+	2						
3	rr G1547	0	0	+	0	+	0	+		+	0	+	0	+	0	+	0	+	0	+	+	+	+	+	0	0	+	+	3						

Underlying autoantibodies were ruled out by auto-control (reaction of patient cells with serum). No statistically significant number of cases tested positive, with a p-value of less than 0.418, with a 95% confidence interval. Autoantibodies were present in two patients out of 140, which is 1.42% of the study population. Alloantibodies occurred in three patients (2.14%). Pan-agglutination came in one sample, so the antibody identification is undetermined for one patient, which is 0.71% of the total study population (Table 2).

**Table 2:** Results of RBC antibody screening.

Results of antibody screening	Result	Number	Percentage
Antibody screening	Positive	5	3.57%
	Negative	135	96.43%
Distribution of antibody	Autoantibodies	2	1.42%
	Alloantibodies	3	2.14%
Frequency of alloantibodies	Anti-c	1	0.71%
	Anti-e	1	0.71%
	Pan-agglutination (antibody not determined)	1	0.71%

Out of 140 samples tested for antibodies, five showed positive results in antibody screening, which is 3.57% of the total study sample, two of which were positive for autoantibodies as the auto-control was positive, and the rest three were alloantibodies. The autoantibodies in the patient had a primary diagnosis of SCD from whom the sample was drawn. The transfusion history of these two patients was known, but the duration since the last donation was not known. Although detailed transfusion intervals were not available for all patients, those with antibodies had a history of multiple prior transfusions, supporting transfusion exposure as an important risk factor. Adsorption elution detection for antibodies was not performed as all sample was used for repeated testing for antibody screening and 16-cell panel antibody identification, but all the panel cells were negative for any antibody in serum at 37°C.

On identification using a 16-cell panel, one showed anti-c and one showed anti-e positivity out of five positive samples for antibody screening, which are 20% and 20% positive cases for antibody screening in this study.

**Figure 3:**
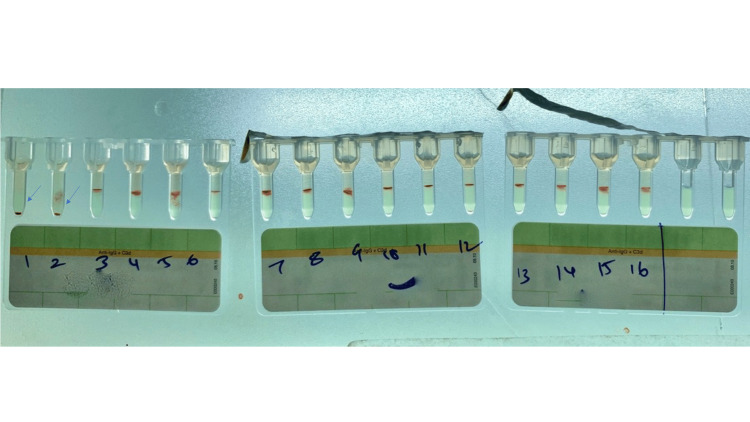
Results for antibody identification using 16-cell panel (Panocell): microtubes 1 and 2 showing negative reaction (arrow) and microtubes 3-16 showing positive reaction.

Out of the total positive cases, two samples (40%) exhibited weak (1+) positivity for autoantibodies, while one sample (20%) demonstrated pan-agglutination (Table 3 and Figure 3).

**Table 3:** Antigram of 16-cell antibody identification panel. * Indicates those antigens whose presence or absence may have been determined using only a single example of a specific antibody. An antigen designated with a "w" represents a weakened expression of the antigen that may not react with all examples of the corresponding antibody. In those instances where a patient's serum is known to contain anti-D, it may be desirable to perform antibody screening tests with D-red cells. The panel cells whose vial numbers are set off by brackets (0) can be used together to form a three- or four-vial D-negative antibody screening reagent. All bracketed cells must be used to construct a complete screening reagent.

			Rh-Hr							Kell						Duffy		Kidd		Lewis		P	MN				Lutheran		Xg	Patient’s test results						
CELL	Special type	Donor	D	C	c	E	e	Cw	K		k	Kpa	Kpb	Jsa	Jsb	Fya	Fyb	Jka	Jkb	Lea	Leb	P1	M	N	S	s	Lua	Lub	Xga	CELL						
1	Bg(a+)	R1R1 B10269	+	+	0	0	+	0	0		+	0	+	0	+	+	0	+	+	+	0	+	+	0	+	0	0	+	0	1						
2		R1wR1 B9287	+	+	0	0	+	+	+		+	0	+	0	+	+	+	0	+	0	+	0	+	+	+	+	+	+	+	2						
3		R2R2 C6359	+	0	+	+	0	0	0		+	0	+	0	+	0	+	+	0	0	+	0	+	0	+	0	0	+	+	3						
4	Go(a+)	Ror D450	+	0	+	0	+	0	0		+	0	+	+	+	0	0	+	+	0	+	+	0	+	0	+	0	+	0	4						
5		r’r E817	0	+	+	0	+	0	+		+	0	+	0	+	+	W	+	+	0	0	0	+	+	+	0	0	+	+	5						
6		r”r F951	0	0	+	+	+	0	0		+	0	+	0	+	+	+	+	0	+	0	+	+	+	0	+	0	+	0	6						
7		rr G1918	0	0	+	0	+	0	+		+	0	+	0	+	0	+	+	0	0	+	0	+	+	0	+	0	+	+	7						
8		rr H1433	0	0	+	0	+	0	0		+	0	+	0	+	+	0	+	0	0	+	+	+	+	+	+	0	+	+	8						
9	Di(a+)	R1R2 A4396	+	+	+	+	+	0	0		+	0	+	0	+	0	+	+	+	0	+	+	+	0	+	0	0	+	0	9						
10		R1R1 B9662	+	+	0	0	+	0	0		+	0	+	0	+	+	0	+	+	+	+	0	0	+	0	+	0	+	0	10						
11		R2R2 C5242	+	0	+	+	0	0	0		+	0	+	0	+	+	+	+	0	0	+	0	0	+	0	+	+	+	+	11						
12		rr H1443	0	0	+	0	+	0	0		+	0	+	0	+	+	0	0	+	+	0	+	+	+	+	+	0	+	0	12						
13		rr N2959	0	0	+	0	+	0	0		+	0	+	0	+	0	+	0	+	0	+	0	+	0	+	0	0	+	+	13						
14		rr N4804	0	0	+	0	+	0	0		+	+	+	0	+	+	+	+	0	+	0	+	0	+	0	+	0	+	+	14						
15		rr G1497	0	0	+	0	+	0	+		+	0	+	0	+	+	0	0	+	0	0	+	0	+	0	+	+	+	+	15						
16	Co(b+)	rr N4291	0	0	+	0	+	0	0		+	0	+	0	+	0	+	0	+	+	0	+	+	0	0	+	0	+	0	16						

This pan-agglutination may have been influenced by the patient's underlying clinical conditions, including multiple transfusions or co-infections, which could have led to the presence of cross-reacting antibodies or antibodies against high-frequency antigens, especially since the auto-control for this case was negative. The patient was diagnosed with SCD accompanied by sepsis and a urinary tract infection. The specific antibody responsible for the reaction was not identified through procedures involving autoadsorption or alloadsorption of red blood cells.

Blood group and positive antibody screening are not statistically associated, as the p-value is 0.319. Most of the patients studied were from the Chhattisgarh region only, and because of genetic homogenicity in the population, the rate of alloimmunization might be on the lower side. Out of five positive cases for antibody screening, two were of "A" blood group, one was having "AB" blood group, and two were having "O" blood group (Table 4).

**Table 4:** Blood group (ABO) and positive antibody screening.

Blood group (ABO)	Negative	Positive
A	32	2
AB	10	1
B	51	0
O	42	2
Total	135	5

## Discussion

Alloimmunization is more common in SCD, with prevalence rates ranging from 7% to 59%, compared to just 2% to 3% in sporadically transfused patients from general hospital populations [9-12]. Global literature on alloimmunization in multi-transfused SCD patients shows a rate of alloimmunization from 2.6% up to 47% [19,23]. Various studies conducted in India have shown that the development of alloantibodies in patients receiving multiple transfusions, such as those with thalassemia, chronic renal failure, surgical oncology, and various other conditions, exhibits a prevalence that varies significantly, ranging from 0.49% to 18.8% [16]. In our study, the rate of alloimmunization in sickle cell patients came to be 2.1%, which is on the lower side compared to rates reported in previous global studies, but it is within the range of multi-transfused patients with diverse clinical diagnoses. Jariwala et al. investigated alloimmunization rates in SCD and β-thalassemia major patients receiving red cell transfusions at Surat Raktadan Kendra and Research Centre, Surat, India, from 2008 to 2016. Their study of 138 patients found an alloimmunization rate of 10.86%, with identified antibodies against c, E, Kell, Jka, and Jkb [16]. In the present study of 140 cases, 2.14% of patients were found to be alloimmunized, indicating a low prevalence in the studied population.

One study compared the transfusion practice for SCD patients between a center in India and a sickle cell management center in Jamaica [24]. The majority of the data on alloantibody formation from Western countries in SCD patients receiving transfusion showed up to 76% alloimmunization, and the prevalence across the world varied between 2.6% in Jamaica to 65% for ABO, RhD only matched red cells in Kuwait, and 76% in Jamaican SCD patients taking transfusion in the UK [25-27]. The increased prevalence of alloantibodies in multi-transfused SCD patients in Western countries can be explained on the basis of racial differences of major red cell antigen systems between blood-donating Caucasian populations and SCD patients from African and Indian-Arab populations. The same argument was given by Moreira et al. for the Brazilian SCD population [25,27,28].

The majority of the studies on alloimmunization in SCD patients have shown that alloantibodies are formed against broad Rh system antigens, e.g., c, C, e, E, D, Kell, and Duffy antigens [29]. A study on alloimmunization by Sarnaik et al. in 1986 on 245 subjects found the rate of alloimmunization to be 7.75%, with antibodies directed against Rh, Kell, and Lea/Leb [25]. That is why it has been suggested that partial phenotyping of patients involving the Rh, Kell, and Duffy antigens may cut down the alloimmunization rate by at least 60-80% [10]. Similarly, in our study, “anti-c” (0.71%) and “anti-e’’ (0.71%) were detected, both directed against Rh antigens, in concordance with older studies.

To mitigate alloimmunization, partial phenotypic matching for Rh (C, c, E, e) and Kell antigens should be prioritized, as these are the most immunogenic and most frequently implicated in clinically significant antibodies in our population. Extending matching to additional antigen systems such as Duffy and Kidd may provide further protection, though this may not be feasible in resource-limited settings. A practical approach is to combine extended antibody screening with targeted phenotypic matching for these high-risk antigens, supported by transfusion record-keeping and antibody registries.

The findings of this study provide evidence that patients with SCD who receive multiple blood transfusions can develop alloantibodies against RBC antigens; however, not all patients develop these alloantibodies after exposure to transfused RBCs. The observed alloimmunization rate in our study is lower compared to Western data, which may be attributed to the genetic homogeneity between donors and SCD patients, as well as the common ethnicity within the population. Based on the antibody profile detected in this study, partial phenotypic matching for Rh (C, c, E, e) and Kell antigens should be prioritized, as these are the most immunogenic and most frequently implicated in clinically significant alloantibodies. Extending matching to other antigens, such as Duffy and Kidd, may provide additional protection, although this may not be feasible in resource-limited settings. As a practical alternative, conducting a complete crossmatch prior to RBC transfusion can help detect clinically significant antibodies. Additionally, screening SCD patients for RBC antibodies and providing antigen-negative blood for known alloimmunized patients can significantly enhance transfusion safety. For acute situations, a database of antigen typing should be recorded electronically to provide whole blood or red cell concentrate in time. Findings of this study provide evidence that patients with SCD receiving blood transfusions are burdened with the development of RBC alloantibodies, although at a lower prevalence compared to well-resourced countries where transfusions are given more frequently. However, in resource-limited settings such as ours, implementation of RBC antigen matching for all patients would be difficult.

Limitations of the study

This study has several limitations. The relatively small sample size and the predominance of participants from local populations may limit the wider use of the findings. In addition, incomplete transfusion histories restricted our ability to evaluate cumulative exposure as a determinant of alloimmunization risk. Many patients may have received recent transfusions, which can interfere with antibody identification due to mixed-field reactions. Finally, as this was a single-center study, variations in transfusion practices across different institutions were not captured. We were unable to perform detailed subgroup analyses by age, transfusion frequency, or duration since last transfusion, and clinical status such as comorbidities or treatment stage, which could have helped identify patterns or additional risk factors for alloimmunization. Future multicentric studies with a larger and more diverse patient population, and with standardized recording of transfusion histories, are warranted to enhance external validity.

## Conclusions

Taken together, the data show that while the overall prevalence of alloimmunization in this population is relatively low (2.14%), the detection of both alloantibodies and autoantibodies underscores the importance of considering a broader antibody spectrum when planning transfusion strategies for SCD patients. To the best of our knowledge, this is the first study of its kind from Chhattisgarh, though alloimmunization has been studied in other parts of India. The alloimmunization rate of 2.14% observed in our cohort confirms that repeated transfusion can lead to clinically significant antibodies in SCD patients, even in populations with relatively homogeneous donor-recipient profiles. Given the antibody profile identified in this study (anti-c and anti-e), partial phenotypic matching for Rh antigens (C, c, E, e) and Kell is advisable, as these are highly immunogenic and commonly implicated in clinically significant alloimmunization. Extending matching to additional antigens, such as Duffy and Kidd, could further decrease risk, although this may be challenging in resource-limited settings. Implementing partial phenotypic matching in transfusion practice would therefore help reduce the likelihood of alloimmunization in SCD patients. Therefore, transfusing partially phenotypically matched RBCs, particularly for Rh (C, c, E, e) and Kell antigens, should be implemented where feasible to reduce this risk.

## Appendices

Detailed procedure for carrying out all screening tests

ABO and Rh Grouping

Introduction: As part of routine transfusion care, ABO and Rh typing is performed on all selected sickle cell (SCD) disease patients. At our center, ABO and Rh grouping was performed by the conventional test, i.e., tube technique for blood groups ABO and RhD, along with reverse grouping. If any grouping discrepancy was observed, a further workup was carried out to resolve the discrepancy.

Materials required: (1) Anti-A, anti-B, anti-D, anti-A,B monoclonal antisera, anti-A1 lectin (ERYCLONE) or ABD gel card (Bio-Rad Ltd). (2) Normal saline (0.9%) or low-ionic strength solution (LISS) as suspending medium (Bio-Rad Ltd). (3) 3-5% suspension of group A, B, and O pooled RBCs for reverse grouping. (4) Test tubes. (5) Tabletop centrifuge (Eppendorf, Hamburg, Germany) or Bio-Rad Gel card centrifuge. (6) Calibrated pipette (0-100 µm) variable volume.

Procedure for blood grouping (test tube method): (1) A sample for grouping was collected in a 3 ml EDTA tube and centrifuged to separate the cells from plasma. (2) Clean test tubes 10x75 mm. (3) A 3-5% suspension of the RBCs was made using normal saline. (4) Two drops of antisera A, B, D, and AB are taken in the corresponding labeled test tubes. (5) 100 μl of separated plasma is taken in test tubes labeled A-cell, B-cell, and O-cell for reverse grouping. (6) 50 μl of the patient’s RBC suspension was put into each test tube with antisera using calibrated pipettes. (7) 50 μl of the pooled A, B, and O cells were put into the test tube labeled A-cell, B-cell, and O-cell for reverse grouping. (8) The tubes were immediately centrifuged in a centrifuge as per the manufacturer’s instructions (for 1 min at 1000 RPM). (9) The test tube is taken out of the centrifuge and examined for agglutination, and the blood group is determined. (10) In case the blood group of the patient was found to be A or AB, the patient was further evaluated for the A subgroups using A1 lectin.

Direct Antihuman Globulin Testing (DAT)

Principle: The direct antihuman globulin testing (DAT) was developed by Coombs, Mourant, and Race for the detection of antibodies attached to red cells that do not produce direct agglutination. DAT is an important serologic test performed using an anti-IgG reagent. A positive test result indicates that the antibody is coating the RBCs.

Materials required for DAT using a gel card: (1) Coombs Gel Card (Bio-Rad Ltd.). (2) Pasteur pipettes. (3) Pipettor tips. (4) Suspension tubes. (5) Incubator at 37°C. (6) Centrifuge for gel card.

Procedure for DAT: (1) The gel card is marked with the unique patient or donor number for identification. (2) A 0.8-1% red cell suspension is prepared from the patient’s RBCs. (3) 50 μl of the 0.8% red cell suspension is pipetted to the microtube wells. (4) Gel card is immediately centrifuged for 10 minutes at 1031 RPM for 10 minutes. (5) Results are read and recorded.

Indirect Anti-Human Globulin Test by Gel Column Agglutination Technique

Principle: The antibody screen test is used in the detection of unexpected blood group antibodies. In this test, the reagent red blood cells in a hypotonic buffered saline solution (LISS) are combined with patient serum/plasma to allow antigen/antibody interaction in the upper chamber of the microtube. This results in promoting antibody uptake. The detection of this antibody occurs when the sensitized red blood cells react with the anti-IgG gel in the microtube during centrifugation.

Materials required: Test cells for antibody screening (Panoscreen® I, II, and III, Immucor, Inc., Norcross, GA). (1) LISS ID-Diluent 2 (Bio-Rad), a hypotonic buffered saline solution. (2) Antibody screening 16-cell panel (PANOCELL 16, Immucor, Inc.) (with appropriate dilution for gel card technique). (3) Anti-IgG+ C3d card, suspended in gel (Bio-Rad). (4) Dry incubator (Bio-Rad). (5)Test tubes (10 x 75 mm). (6) Tabletop gel card centrifuge (Bio-Rad). (7) Calibrated pipette.

Procedure for column agglutination technique: (1) Coombs gel cards were labeled with the appropriate patient identification and test information (I, II, and III). (2) The foil seal from the card was removed. (3) Using an appropriate pipette, 50 mL of each antibody screen red blood cell (0.8%-1.0%) suspension was added to the corresponding microtube. (4) Using an appropriate pipette, 25 mL of plasma was added to each of the three microtube wells. (5) The gel card was incubated at 37°C for 15 minutes in a dry incubator (Bio-Rad). (6) Following incubation, the gel cards were centrifuged for 10 minutes inside a tabletop gel card centrifuge (Bio-Rad) at a fixed RPM (1031), as per the manufacturer’s instructions. (7) The front and the back of each microtube were read visually, and reactions were recorded as described in the Interpretation section below. The data sheet of patients who tested positive by this method only is provided below.

Interpretation:

I. Negative: A complete sedimentation of all red cells on the bottom of the microtube.

II. 4+ grade reaction (strong positive): Cells remain on the top of the gel.

III. 3+ grade reaction: Agglutinated RBCs are suspended till the upper half of the gel column.

IV. 2+ grade reaction: Agglutinated RBCs are dispersed throughout the gel.

V. 1+ grade reaction: Agglutinated RBCs are present only in the lower half of the column.

VI. Mixed cell reactions may be recognized as a layer of positive cells at the top of the gel, accompanied by a button of negative cells at the bottom of the microtube.

VII. Clots, particles, or other artefacts may cause some cells to be entrapped at the top of the gel, which may cause an anomalous result in a negative test.

Antibody screening steps: (1) Rule out common antibodies from all the negative reactions. (2) Use the double-dose reaction to rule out antibodies showing dosage. (3) Shortlist the most probable antibodies from the list of antibodies if the reaction is seen in more than one panel of cells. (4) The confirmation is to be made using the extended panel of cells or specific antiserum as applicable.

Identification of Antibodies Using 16-Cell Antibody Identification Panel

Principle: Once an antibody has been detected, additional testing is necessary to identify the antibody and determine its clinical significance. The patient’s serum or plasma is tested against additional RBCs possessing known antigens. The test method used should be as sensitive as that used for detection. An antibody identification panel is a collection of 11 or 16 group O RBCs with various antigen expressions. The pattern of antigen expression should be diverse so that it will be possible to distinguish one antibody from another and should at least include cells with homozygous expression of Rh subgroups, Duffy, Kidd, and MNS antigens.

Materials required: (1) PANOCELL 16 reagent cell panel of test cells (Immucor, Inc.). (2) LISS ID-Diluent 2 (a hypotonic buffered saline solution) (Bio-Rad). (3) Dry incubator (Bio-Rad). (4) Test tubes. (5) Tabletop gel card centrifuge (Bio-Rad). (6) Calibrated pipettes. (7) Pasteur pipettes. (8) Antegram (a profile sheet specifying the antigens on each panel).

Procedure: (1) The Coombs gel card columns were labeled with the appropriate cell number (1 to 11). (2) The foil seal was removed from the card. Using an appropriate pipette, 50 μL of each antibody screen red blood cell suspension was added to the properly labeled correct gel microtube. (3) Using an appropriate pipette, 25 μL of plasma was added to all the micro columns. (4) Incubated at 37°C for 15 minutes in a dry incubator. (5) The gel cards were centrifuged as per the manufacturer’s instructions (at 1031 RPM for 10 minutes). (6) The front and the back of each microtube were read macroscopically, and reactions were recorded for interpretation as given below.

Interpretation: (1) Rule out common antibodies from all the negative reactions. (2) Use the double-dose reaction to rule out antibodies showing dosage. (3) Shortlist the most probable antibodies from the list of antibodies if the reaction is seen in more than one panel of cells (rule-in). (4) The confirmation is to be made using the selected cells or specific antiserum, as applicable. (5) Use the dictum of three (minimum two) antibody-positive reagent cells and three (minimum two) antibody-negative reagent red cells for shortlisting the implicated antibodies. (6) Confirm the antibody using the antibody-specific reagents (antiserum) with appropriate positive and negative controls.

The interpretation was conducted using the “Rule-out” technique, in accordance with the recommended method. The initial step in interpreting panel results involved excluding antibodies that could not account for the observed reactivity. To carry out these exclusions, RBCs that exhibited a negative reaction throughout all testing phases were analyzed. Once each negatively reacting cell was assessed, the remaining antigens were evaluated to determine if their reactivity patterns corresponded with patterns of antigen-positive cells (inclusion technique). The evaluation of panel results followed a systematic approach to ensure accurate identification and to prevent overlooking specific antibody characteristics that could be obscured by other antibodies. For instance, a positive reaction noted with cell panels 1, 2, 3, and 8 identified the corresponding antibody as anti-D, based on the antigram provided with the reagent cell panel. Similarly, a positive reaction with cell panels 1, 2, 3, 4, and 8 indicated a combination of anti(C + D) through the panel interpretation.

Adsorption Procedure and Elution Procedure

1. Rinse the selected red cells or reagent cells at least three times with saline.

2. After the final wash, centrifuge the red cells at 800 to 1000 × g for a minimum of five minutes, and remove as much supernatant saline as possible. Any remaining saline can be absorbed by briefly touching the red cell mass with a narrow strip of filter paper.

3. Mix suitable volumes of the packed red cells and serum, then incubate at the desired temperature for 30 to 60 minutes.

4. Stir the serum or cell mixture periodically during the incubation period.

5. Centrifuge the red cells at 800 to 1000 × g for five minutes to compact the cells tightly. If possible, perform centrifugation at the incubation temperature to minimize antibody dissociation from the red cell membranes.

6. Transfer the supernatant fluid, which is the adsorbed serum, to a clean test tube. If preparing an eluate, retain the red cells.

7. Test an aliquot of the adsorbed serum against a reserved, unused aliquot of the red cells or reagent cells with a known antigenic profile used for adsorption to confirm that all antibodies have been removed.

Heat Elution Procedure

1. Rinse the patient’s or adsorbed red cells five to six times with normal saline (0.9%).

2. Utilize the supernatant from the final wash as a negative control for the adsorbed antibody.

3. Combine equal volumes of washed, packed cells and 6% bovine albumin in a 13 × 100 mm test tube.

4. Incubate the tube at 56°C for 10 minutes, agitating it periodically throughout the incubation.

5. Centrifuge the tube at 900 to 1000 × g for two to three minutes.

6. Carefully transfer the supernatant eluate into a clean test tube, and test it alongside the supernatant saline from the final wash.

7. Confirm the presence of antibodies using a commercial or in-house three-cell panel, followed by an extended panel for further verification.

This proposal has been reviewed and approved by the institutional ethics committee (IEC), which is a committee whose task it is to make sure that research participants are protected from harm.

**Table 5:** Master chart. Hb: hemoglobin; HX: history; HPLC: high-performance liquid chromatography; M: male; F: female; AKI: acute kidney injury; SC: sickle cell; SCD: sickle cell disease; UTI: urinary tract infection; TB: tuberculosis; HCT: hematocrit; SDH: subdural hemorrhage; SAH: subarachnoid hemorrhage; SCA: sickle cell anemia; VOC: vaso-occlusive crisis; CCF: congestive cardiac failure; CLD: chronic liver disease; MODS: multiple organ dysfunction syndrome; RHD: rheumatic heart disease; MS: mitral stenosis; VWD: von Willebrand disease; TTP: thrombotic thrombocytopenic purpura; ICT: indirect Coombs test; CKD: chronic kidney disease; ALL: acute lymphoblastic leukemia.

S. No.	Sample No.	Age (year)	Gender	Blood group (ABO)	Rh	History of transfusion	Hb	HCT	Clinical HX	Antibody screening	Antibody identified	HPLC
1	Sample 1	6	M	A	Positive	Yes	4.8	15	SCD with dyskeratosis congenita	Negative	0	NA
2	Sample 2	10	M	B	Positive	Yes	3.6	11.5	SCD with SDH	Negative	0	Yes
3	Sample 3	23	F	O	Positive	Yes	5.1	16.3	SCD	Positive	c	Yes
4	Sample 4	18	F	O	Positive	Yes	6.7	29.1	SCD with AKI	Negative	0	Yes
5	Sample 5	27	F	O	Positive	Yes	5.3	17.1	SCD	Negative	0	Yes
6	Sample 6	6	M	A	Positive	Yes	1.7	5.1	SCD with dyskeratosis congenita	Negative	0	Yes
7	Sample 7	19	M	A	Positive	Yes	6.5	19.5	SCD	Negative	0	Yes
8	Sample 8	29	M	A	Positive	Yes	7	21	SCD	Negative	0	Yes
9	Sample 9	35	F	O	Positive	Yes	6.4	23.1	Cushing disease with SCD	Negative	0	Yes
10	Sample 10	19	M	A	Positive	Yes	7.5	22.5	SCD	Negative	0	Yes
11	Sample 11	16	F	O	Positive	Yes	7	21	SCD	Negative	0	Yes
12	Sample 12	47	M	O	Positive	Yes	2.8	20.4	SCD	Negative	0	Yes
13	Sample 13	13	F	B	Positive	Yes	11.5	34.5	SCD	Negative	0	Yes
14	Sample 14	20	M	O	Positive	Yes	6.9	20.7	SCD	Negative	0	Yes
15	Sample 15	24	M	O	Positive	Yes	7.9	23.7	SCD	Negative	0	Yes
16	Sample 16	24	F	O	Positive	Yes	6.7	20	SCD	Negative	0	Yes
17	Sample 17	19	M	A	Positive	Yes	6.5	19.6	SCD	Negative	0	Yes
18	Sample 18	25	M	AB	Negative	Yes	5.1	14.7	SCD	Positive	e	Yes
19	Sample 19	9	F	B	Positive	Yes	7.5	23.7	SCD with SAH	Negative	0	Yes
20	Sample 20	50	F	B	Positive	Yes	8.4	23.2	SCD with sepsis	Negative	0	Yes
21	Sample 21	52	F	B	Positive	Yes	7.6	21.2	SCD with sepsis	Negative	0	Yes
22	Sample 22	17	M	A	Positive	Yes	8.4	23.2	SCD with # Lt. femur	Negative	0	Yes
23	Sample 23	55	F	B	Positive	Yes	3.6	10.3	SCD with sepsis	Negative	0	Yes
24	Sample 24	17	M	B	Positive	Yes	7.3	21.9	SCD	Negative	0	Yes
25	Sample 25	40	M	B	Positive	Yes	4.9	14.7	SCA with VOC	Negative	0	Yes
26	Sample 26	23	M	AB	Positive	Yes	6.6	19.8	SCD	Negative	0	Yes
27	Sample 27	60	F	B	Positive	Yes	11	33	SCD	Negative	0	Yes
28	Sample 28	24	F	B	Positive	Yes	6.5	19.5	SCD	Negative	0	Yes
29	Sample 29	17	M	B	Positive	Yes	6.6	19.8	SCD	Negative	0	Yes
30	Sample 30	40	M	B	Positive	Yes	7.1	21.3	SCD	Negative	0	Yes
31	Sample 31	17	M	B	Positive	Yes	2.8	7.4	SCD	Negative	0	Yes
32	Sample 32	27	M	B	Positive	Yes	2.9	8.6	SCD	Negative	0	Yes
33	Sample 33	23	M	B	Positive	Yes	7.6	22.2	SCD	Negative	0	Yes
34	Sample 34	40	M	B	Positive	Yes	4.9	14.2	SCD with VOC	Negative	0	Yes
35	Sample 35	18	M	B	Positive	Yes	3.5	10.8	SCD with VOC	Negative	0	Yes
36	Sample 36	48	F	O	Positive	Yes	6.6	19.8	SCD	Negative	0	Yes
37	Sample 37	50	F	O	Positive	Yes	8.4	25.2	SCD	Negative	0	Yes
38	Sample 38	34	F	O	Positive	Yes	4	12	SCD	Negative	0	Yes
39	Sample 39	19	M	B	Positive	Yes	10.6	31.8	SCD	Negative	0	Yes
40	Sample 40	60	M	O	Positive	Yes	5.2	15.6	SCD	Negative	0	Yes
41	Sample 41	18	M	B	Positive	Yes	5.6	16.8	SCD	Negative	0	Yes
42	Sample 42	21	M	B	Positive	Yes	5.5	15.5	SCD with VOC	Negative	0	Yes
43	Sample 43	23	M	B	Positive	Yes	6	18	SCD with VOC	Negative	0	Yes
44	Sample 44	27	F	B	Positive	Yes	8.7	25.1	SCD	Negative	0	Yes
45	Sample 45	18	M	B	Positive	Yes	5.6	16.8	SCD	Negative	0	Yes
46	Sample 46	7	M	AB	Positive	Yes	5.6	16.8	SCD with pneumonia	Negative	0	Yes
47	Sample 47	5	F	AB	Positive	Yes	3.7	10.1	SCD with VOC	Negative	0	Yes
48	Sample 48	50	F	O	Positive	Yes	4.3	12.9	SCD with septic encephalitis	Negative	0	Yes
49	Sample 49	58	F	O	Positive	Yes	5	15	SCD with septic encephalitis	Negative	0	Yes
50	Sample 50	34	F	B	Positive	Yes	4.8	14.4	SC trait with nephrotic syndrome	Negative	0	Yes
51	Sample 51	40	M	B	Positive	Yes	9.2	27	SCD	Negative	0	Yes
52	Sample 52	32	M	A	Positive	Yes	7.6	22.8	SCD with nephrotic syndrome	Negative	0	Yes
53	Sample 53	33	M	A	Positive	Yes	5.6	16.8	SCD	Negative	0	Yes
54	Sample 54	46	F	B	Positive	Yes	4.8	13.4	SCD with CCF	Negative	0	Yes
55	Sample 55	46	F	B	Positive	Yes	5	15	SCD with CCF	Negative	0	Yes
56	Sample 56	44	F	B	Positive	Yes	4.9	1.7	SCD	Negative	0	Yes
57	Sample 57	19	M	A	Positive	Yes	7.1	21.3	SCD with hepatosplenomegaly	Positive	Mixed field	Yes
58	Sample 58	28	F	A	Positive	Yes	4	12	SCD with hypothyroidism	Negative	0	Yes
59	Sample 59	18	M	O	Positive	Yes	3.6	10.8	SCD with CLD	Negative	0	Yes
60	Sample 60	28	F	A	Positive	Yes	7.8	22.4	SCD hypothyroidism	Negative	0	Yes
61	Sample 61	18	F	O	Positive	Yes	4	13	SCD hypothyroidism	Negative	0	Yes
62	Sample 62	23	F	O	Positive	Yes	7	21	SCD hypothyroidism	Negative	0	Yes
63	Sample 63	36	M	B	Positive	Yes	5.1	15.5	SC trait with MODS, AKI	Negative	0	Yes
64	Sample 64	19	M	A	Positive	Yes	3	9	SCD with CLD	Negative	0	Yes
65	Sample 65	22	F	A	Positive	Yes	14.3	42.1	SCD with RHD, MS	Negative	0	Yes
66	Sample 66	26	M	B	Positive	Yes	2.8	7.4	SCD with VWD	Negative	0	Yes
67	Sample 67	26	M	B	Positive	Yes	3.5	13.5	SCD with VWD	Negative	0	Yes
68	Sample 68	24	M	B	Positive	Yes	7	20.5	SCD with VWD	Negative	0	Yes
69	Sample 69	55	F	O	Positive	Yes	6.3	18.8	SCD	Negative	0	Yes
70	Sample 70	33	F	O	Positive	Yes	3	8.8	SCD	Negative	0	Yes
71	Sample 71	52	M	O	Positive	Yes	3.5	14.2	SCD with CLD	Negative	0	Yes
72	Sample 72	5	M	B	Positive	Yes	9.9	28.5	SCD with TTP with renal failure	Negative	0	Yes
73	Sample 73	9	F	B	Positive	Yes	2.6	7.5	SCD	Negative	0	Yes
74	Sample 74	52	M	O	Positive	Yes	4.8	17.2	SCD with CLD	Negative	0	Yes
75	Sample 75	25	M	O	Positive	Yes	6.1	18	SCD with CLD	Negative	0	Yes
76	Sample 76	27	F	A	Positive	Yes	7.4	22	SCD in labor	Negative	0	Yes
77	Sample 77	34	F	A	Positive	Yes	7.7	23	SCD in labor	Negative	0	Yes
78	Sample 78	19	M	A	Positive	Yes	7.4	21.5	SCD	Negative	0	Yes
79	Sample 79	15	M	A	Positive	Yes	3.2	12.2	SCD with pancytopenia	Negative	0	Yes
80	Sample 80	23	M	B	Positive	Yes	6.4	19.2	SCD with bilateral pyothorax	Negative	0	Yes
81	Sample 81	27	F	A	Positive	Yes	7.7	23.1	SCD	Negative	0	Yes
82	Sample 82	47	M	B	Positive	Yes	7	21.2	SCD	Negative	0	Yes
83	Sample 83	5	F	B	Positive	Yes	7.3	24.3	SCD	Negative	0	Yes
84	Sample 84	40	F	B	Positive	Yes	4.8	14.4	SCD with cholelithiasis	Negative	0	Yes
85	Sample 85	3	F	B	Positive	Yes	4.3	14.8	SCD	Negative	0	Yes
86	Sample 86	22	M	O	Positive	Yes	6.3	18.9	SCD	Negative	0	Yes
87	Sample 87	17	M	O	Positive	Yes	7.4	21.8	SCD	Negative	0	Yes
88	Sample 88	14	F	A	Positive	Yes	8	23.5	SCD	Negative	0	Yes
89	Sample 89	48	F	B	Positive	Yes	3.8	11.3	SCD with pancytopenia	Negative	0	Yes
90	Sample 90	6	F	AB	Positive	Yes	5.9	17	SCD	Negative	0	Yes
91	Sample 91	35	M	B	Positive	Yes	8.7	26	SCD in SC crisis	Negative	0	Yes
92	Sample 92	30	M	A	Positive	Yes	6.2	18.1	SCD	Negative	0	Yes
93	Sample 93	15	F	B	Positive	Yes	5.8	17.2	SCD	Negative	0	Yes
94	Sample 94	23	F	A	Positive	Yes	3.9	11.7	SCD	Positive	Autoantibody	Yes
95	Sample 95	16	F	A	Positive	Yes	4	12.3	SCD	Negative	0	Yes
96	Sample 96	54	F	A	Positive	Yes	5.7	17.5	SCD	Negative	0	Yes
97	Sample 97	4	F	O	Positive	Yes	3.8	11.2	SCD	Negative	0	Yes
98	Sample 98	24	F	O	Positive	Yes	4	12	SCD	Negative	0	Yes
99	Sample 99	17	M	O	Positive	Yes	5.6	17.8	SCD with VOC	Negative	0	Yes
100	Sample 100	20	M	AB	Positive	Yes	5	15	SCD	Negative	0	Yes
101	Sample 101	34	M	AB	Positive	Yes	5.8	17.5	SCD	Negative	0	Yes
102	Sample 102	54	M	AB	Positive	Yes	6.7	20	SCD	Negative	0	Yes
103	Sample 103	44	M	AB	Positive	Yes	7	21	SCD	Negative	0	Yes
104	Sample 104	11	F	A	Positive	Yes	4.4	12.5	SCD	Negative	0	Yes
105	Sample 105	13	F	A	Positive	Yes	5.3	15	SCD	Negative	0	Yes
106	Sample 106	24	F	A	Positive	Yes	6.4	19.2	SCD	Negative	0	Yes
107	Sample 107	6	M	AB	Positive	Yes	6.6	20.4	SCD with osteomyelitis with pneumonia	Negative	0	Yes
108	Sample 108	22	M	B	Positive	Yes	9.7	29	SCD	Negative	0	Yes
109	Sample 109	54	F	O	Positive	Yes	8.3	24	SCD with hypothyroidism	Negative	0	Yes
110	Sample 110	30	F	A	Positive	Yes	7.7	21.2	SCD	Negative	0	Yes
111	Sample 111	13	M	O	Positive	Yes	7.1	21	SCD with increased ICT	Negative	0	Yes
112	Sample 112	32	M	O	Positive	Yes	2.7	8	SCD with hemolytic crisis	Negative	0	Yes
113	Sample 113	14	M	O	Positive	Yes	4	12	SCA with hemolytic crisis	Negative	0	Yes
114	Sample 114	54	M	O	Positive	Yes	6.2	17	SCA with hemolytic crisis	Negative	0	Yes
115	Sample 115	20	M	O	Positive	Yes	6.2	17	SCA with hemolytic crisis	Negative	0	Yes
116	Sample 116	52	M	B	Positive	Yes	7	20.2	SCD with CKD with uremia	Negative	0	Yes
117	Sample 117	11	M	B	Positive	Yes	7.1	21	SCD with CKD with Uremia	Negative	0	Yes
118	Sample 118	45	F	O	Positive	Yes	6	17	SCA	Negative	0	Yes
119	Sample 119	11	F	A	Positive	Yes	9.9	19.8	SCD	Negative	0	Yes
120	Sample 120	45	F	O	Positive	Yes	6.6	18.2	SCA	Negative	0	Yes
121	Sample 121	39	F	O	Positive	Yes	6	18	SCD with sepsis with UTI	Negative	0	Yes
122	Sample 122	7	F	O	Positive	Yes	3.7	11	SCD with VOC	Negative	0	Yes
123	Sample 123	44	M	B	Negative	Yes	6	18	SCD trait sepsis with UTI	Negative	0	Yes
124	Sample 124	20	M	O	Positive	Yes	6.8	17	SCD in SC crisis	Negative	0	Yes
125	Sample 125	29	M	O	Positive	Yes	6.8	19	SCD	Negative	0	Yes
126	Sample 126	23	F	B	Positive	Yes	6.4	17	SCD with severe anemia	Negative	0	Yes
127	Sample 127	35	M	B	Positive	Yes	3.8	11.2	SCD in hemolytic crisis	Negative	0	Yes
128	Sample 128	22	F	A	Positive	Yes	4.8	14.4	SCA	Negative	0	Yes
129	Sample 129	34	F	A	Positive	Yes	4.9	15	SCA	Negative	0	Yes
130	Sample 130	5	M	O	Positive	Yes	2.9	8.2	SCD with ALL	Negative	0	Yes
131	Sample 131	12	M	O	Positive	Yes	4.2	12.3	SCD with ALL	Negative	0	Yes
132	Sample 132	28	M	AB	Positive	Yes	9	24.3	SCD in SC crisis	Negative	0	Yes
133	Sample 133	27	F	B	Positive	Yes	3.4	10.2	SCD	Negative	0	Yes
134	Sample 134	7	F	O	Positive	Yes	3.4	9.7	SCD	Positive	Autoantibody	Yes
135	Sample 135	12	M	A	Positive	Yes	4.2	12.6	SCD	Negative	0	Yes
136	Sample 136	18	M	O	Positive	Yes	9.1	27.3	SCD with acute chest syndrome	Negative	0	Yes
137	Sample 137	27	F	B	Positive	Yes	6.5	19.5	SCD	Negative	0	Yes
138	Sample 138	9	F	A	Positive	Yes	6.4	19	SCD with TB	Negative	0	Yes
139	Sample 139	11	F	A	Positive	Yes	9.9	30	SCD in SC crisis	Negative	0	Yes
140	Sample 140	26	M	B	Positive	Yes	7.9	20.7	SCD with VOC	Negative	0	Yes
